# Adsorption of phosphate in water using one-step synthesized zirconium-loaded reduced graphene oxide

**DOI:** 10.1038/srep39108

**Published:** 2016-12-15

**Authors:** Xin Luo, Xiurong Wang, Shaopan Bao, Xiawei Liu, Weicheng Zhang, Tao Fang

**Affiliations:** 1 Institute of Hydrobiology, Chinese Academy of Sciences, Wuhan 430072, China; 2 Graduate University of Chinese Academy of Sciences, Beijing 100049, China

## Abstract

In this account, a one-step green hydrothermal method for zirconium-loaded reduced graphene oxide (RGO-Zr) adsorbent was developed in pure water. It is based on the formation of initially strong-coupling RGO-Zr nanocomposites followed by *in situ* reduction of GO to RGO during the hydrothermal treatment. The phosphate adsorption performance of the as-prepared nanocomposites was investigated in aqueous environment under various conditions. The characterization results of RGO-Zr nanocomposites showed that ZrO_2_ was successfully integrated onto the RGO sheets in amorphous. The data from equilibrium phosphate adsorption on RGO-Zr revealed that the adsorption kinetics followed a pseudo-second-order kinetic model, where the adsorption isotherm fitted the Langmuir isotherm model with a maximum adsorption capacity of 27.71 mg P/g at pH 5 and 298 K. The improved phosphate adsorption on RGO-Zr was caused by the dispersion of ZrO_2_ on the RGO surface. Furthermore, the phosphate adsorption was found insensitive to the increase in pH while it was sensitive to the increase in temperature. The coexisting anions of SO_4_^2−^, F^−^, Cl^−^, NO^3−^ and CO_3_^2−^ affected the phosphate adsorption in a different way. Results suggest that the present RGO-Zr adsorbent has the potential for controlling phosphorus pollution in water.

Water eutrophication could lead to several harmful effects, including the destruction of the aquatic life, deterioration of water quality, and even have a hazardous influence on humans^1^,^2^. As a result, removal of excessive phosphorus is important for controlling water eutrophication. A number of methods and techniques have been developed for phosphorus removal, including chemical precipitation, adsorption, and biological treatments^3^,^4^,^5^. Among these available methods, chemical precipitation (with aluminum, iron and calcium salts) caused sludge handling, where the biological treatments were found unsuitable for removal of low phosphate concentrations from the water. The sorption-based methods, however, were demonstrated to be more effective for several advantages, such as low cost, simple operating conditions, stable phosphate removal efficiency, and produce little sludge^6^,^7^,^8^. Recently, the application of low-cost and abundant natural or synthetic materials for phosphate removal has extensively been investigated, including modified bentonites^9^, waste alum sludge^10^, and steel slags^11^.

Nowadays, zirconium based oxides are widely employed for removal of phosphate from water for their chemical stability, non-toxicity, insolubility, resistance to oxidant agents^1^,^6^, and amphoteric character (containing cationic and anionic ion exchange)^12^. Some studies have focused on the modification of adsorbents like ZrO_2_^13^, amorphous zirconium oxide nanoparticles^6^, and amorphous zirconium hydroxide^12^. Others have loaded zirconium based oxides on different materials or metals, such as zirconium-modified zeolites^1^, Fe-Zr binary oxides^14^, zirconium (IV) loaded fibrous adsorbent^4^, zirconia-functionalized graphite oxide^7^, and zirconia-functionalized SBA-15^15^. Although the zirconium based materials have been shown to have a great performance for removal of phosphate, some of them were ultrafine powders or particulates that are hard to separate from the solution. To overcome the problem, the powders required immobilization on suitable substrates.

Carbonaceous based materials, such as activated carbon (AC), carbon nanotubes, porous carbon and graphene oxide, have attracted tremendous interests due to their superior properties, including chemical stability, large specific surface area, abundant pore size distribution, and feasibility for mass production^1^,^16^. Graphene oxide (GO) has a stable layered structure with various oxygen-containing functional groups like hydroxyl, carbonyl, epoxy and carboxylic fragments attached directly to the carbons or at the layers edges^17^,^18^. This results in versatile modified surfaces^7^,^18^. The presence of these various oxygen-containing functional groups on the surface of Graphene oxide (GO) or reduced graphene oxide (RGO) can form complexes with metal ions, such as hydrated zirconium oxide^19^, titanium dioxide^20^, iron oxides^21^, and aluminum oxides^22^. Additionally, GO can be obtained from cheap natural graphite in large quantities and RGO can be collected through reduced processes of GO.

In order to benefit from the advantages of both GO/RGO and zirconium based oxides, a new composite adsorbent was synthesized and characterized. Zong *et al*.^7^ have previously prepared a zirconia-functionalized graphite oxide absorbent, where the composite showed good performance for removal of phosphate. However, toxic anhydrous toluene was used during the synthesis and the reaction was conducted under N_2_ atmosphere, which made the synthetic method more complex and harmful. Therefore, developing alternative green and facile methods is highly desirable. A recent study has reported the successful combination of TiO_2_ onto RGO by hydrothermal method with improved photocatalytic performance of TiO_2_^20^. Hence, the feasibility of applying the hydrothermal method for combination of GO with zirconium-based oxide was investigated in search for novel absorbents.

The objectives of the present paper are: (i) assessment of the practicability of the one-step hydrothermal method in synthesis of zirconium-loaded reduced graphene oxide material, (ii) characterization of the newly prepared materials, (iii) investigation of the adsorption ability of the composite towards phosphate, and (iv) evaluation of several influencing parameters on the phosphate adsorption characteristics, including initial concentrations of phosphate, contact time, pH, temperature, and coexisting anions.

## Methods and Materials

### Synthesis of RGO-Zr

Graphene oxide (GO) was synthesized using a modified Hummers method^7^,^23^. The RGO-Zr (in which Zr is representing zirconium oxides) nanocomposite was obtained through a one-step hydrothermal method. Briefly, a calculated amount of zirconium isopropoxide (Zr(OC_3_H_7_)_4_, Aldrich, 99.99%, 0.065–0.65 g) and GO powder (0.065 g) were added into 30 mL de-ionized water (DI-water 18.2 MΩ/cm; Sartorius Stedim). The mass ratios of GO and zirconium isopropoxide were varied from 1:1 to 1:10 in search for optimized materials. After an ultrasonic dissolution for 0.5 h, the mixture was transferred into a 45 ml Teflon sealed autoclave and maintained at 160 °C for 10 h. For purification, the composite was washed with DI-water for several times then fully freeze dried at −80 °C for 24 h. The Fig. S1 showed that mass ratios of GO: zirconium isopropoxide above 1:6 yielded no increase in the adsorption capacity. Thus, a mass ratio of 1:6 was selected as optimal and used for the synthesis of RGO-Zr in subsequent experiments. All the chemicals used were reagent grade or better.

### Characterization

The structures and properties of GO, RGO-Zr and Zr(OC_3_H_7_)_4_ were characterized by various methods. X-ray diffraction (XRD) patterns were obtained on a D/MAX-RBX-ray diffractometer (Rigaku, Japan) with Cu Kα radiation (λ = 0.15418 nm). Transmission electron microscope (TEM, HT-7700, Hitachi, Japan) was used to analyze the morphology along with Field Emission Scanning Electron Microscopy (FESEM, S-4800, Hitachi, Japan). X-ray photoelectron spectroscopy (XPS) measurements were obtained on an ESCALAB 250Xi (Thermo Fisher) system with Al, Kα sources and all the binding energies were referenced to the C1s peak at 284.8 eV for the surface adventitious carbon. The binding properties were analyzed by Fourier Transform Infrared Nexus spectrophotometer (FTIR, Thermo Nicolet, America). Raman spectra were collected using an INVIA spectrophotometer (Renishaw, UK). The nitrogen adsorption–desorption isotherms were obtained on a Micrometrics ASAP 2020 apparatus at −196 °C. Finally, the surface zeta potentials of the samples were measured using a Zetasizer Nano ZS (Malvern, UK).

### Adsorption studies

The potassium dihydrogen phosphate was used to compound the stock solution and was diluted with DI-water to form various phosphate concentrations. The pH was adjusted to 5.00 ± 0.20 using HCl (0.10 mol/L) and NaOH (0.10 mol/L) solutions. All suspensions flasks were sealed with parafilm and placed in a thermostatic shaker (100 rpm) at 298 K for 24 h. For the equilibrium adsorption isotherm study, the employed adsorbents were RGO-Zr, Zr(OC_3_H_7_)_4_, and GO powders. At low initial levels of phosphate below 0.70 mg/L, the adsorbent dose (0.01 g) and the volume of phosphate solution (400 mL) were constant. At higher initial concentrations of phosphate above 1.00 mg L^−1^, adsorbent (0.04 g) was put in 200 mL solutions. For the kinetics studies of phosphate removal with RGO-Zr, at low initial levels of phosphate (0.10, 0.30, 0.50 mg/L), the adsorbent (0.01 g) was put in 400 mL solutions. At higher initial concentrations of phosphate (1.00, 3.00, 5.00 mg/L), the adsorbent (0.04 g) was suspended in 200 mL solutions. A fixed volume of sample was taken out from the flask at predetermined time intervals.

Unless noted otherwise, the sampling volume and measurement method were performed following the route. First, the sample (3.00 mL) was taken out from the flask. The mixture was then filtered off using 0.45 μm membrane filters and the residual concentration of phosphate was obtained using the molybdenum blue method on a UV-1910 UV/Vis spectrometer at the detection wavelength of 883 nm. All the experiments were performed in duplicate modes and the averages were calculated. The adsorption amount of phosphate was estimated according to equations (1) to (3):


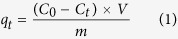



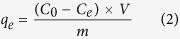



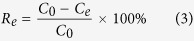


where *q*_*t*_ is the adsorption amount (mg/g) at time *t* (min), *q*_*e*_ is the adsorbed amount (mg/g) of phosphate at equilibrium, *C*_*0*_ is the initial phosphate concentration (mg/L), *C*_*e*_ is the equilibrium concentration (mg/L), *V* is the solution volume (L), *m* is the absorbent mass (g), and *R*_*e*_ represents the removal efficiency at the adsorption equilibrium.

### Effect of pH

The electrolyte pH constitutes one of the most important parameters that control the ion sorption process. The effect of pH on the adsorption of phosphate was evaluated by changing the initial pH from 2.00 to 11.30. This was done through agitation of a 30 mL (V) phosphate solution (*C*_0_ = 5.00 mg/L) put in a 100 mL flask containing 0.005 g of the adsorbent (RGO-Zr), shaken at a rate of 100 rpm for 24 h at 298 K.

### Effect of temperature

The temperature can affect the adsorption process, thus was investigated at 288, 298, 308, and 323 K. The procedure consists of adding 0.005 g adsorbent (RGO-Zr) into a 500 mL flask containing 200 mL phosphate solution at different initial phosphate concentrations (0.10, 0.30, 1.00, and 3.00 mg/L at pH = 5.00), shaken at 100 rpm for 24 h.

### Effect of coexisting anions

To investigate the competing effect of coexisting anions on the adsorption of phosphate, Na_2_SO_4_, NaF, NaCl, NaNO_3_ and Na_2_CO_3_ at concentrations varying from 0 to 5.00 mM were added to the phosphate solutions, respectively. For all experiments, the initial concentration of phosphate was 5.00 mg/L (0.002 mM) and that of the absorbent was 0.20 g/L. All the experiments were conducted in solutions at pH = 5.00 shaken at a rate of 100 rpm for 24 h at 298 K.

## Results and Discussion

### Characterization of adsorbents

**Figure 1:**
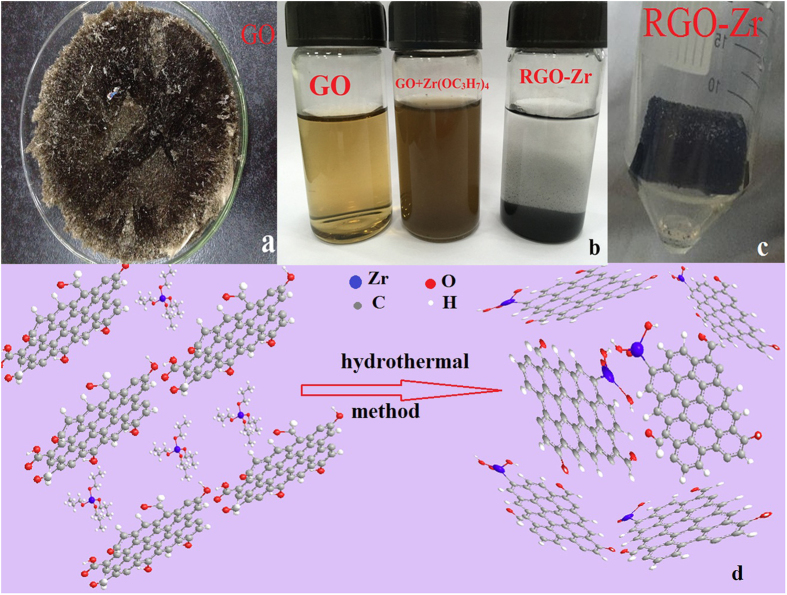
Photographs of (**a**) GO, (**b**) solution of (GO left, a mixture of Zr(OC_3_H_7_)_4_ and GO middle, solution of RGO-Zr right), (**c**) RGO-Zr, and (**d**) the proposed self-assembly and wrapping mechanism for RGO-Zr architecture formation during the hydrothermal treatment.

The GO showed a spongy morphology after the freeze-drying process (Fig. 1a), while when dispersed in water it showed great dispersibility and hydrophilic features (Fig. 1b, left). The newly prepared material formed a three-dimensional structure hydrogel (Fig. 1c) during the hydrothermal treatment without stirring, and the solution was clean and transparent, indicting the efficient embedding zirconium particles. The color of the mixed solution was brown yellow solution (Fig. 1b, middle), however, the color of the suspension of RGO-Zr (Fig. 1b, right, after freeze drying) was black, indicating the reduction of GO to RGO. The reduction mechanism is likely related to H^+^ catalyzed intramolecular and intermolecular dehydration^24^. During the reduction, the number of the oxygenic groups was reduced, and the electrostatic as well as hydrophilic interactions were weakened, graphene oxide nanosheets were self-assembled and rearranged to form a network and the zirconium particles were captured into the network (Fig. 1d). Similar phenomenon was also found in Chen *et al*.^25^, Bai *et al*.^24^, Sheng *et al*.^26^.

**Figure 2:**
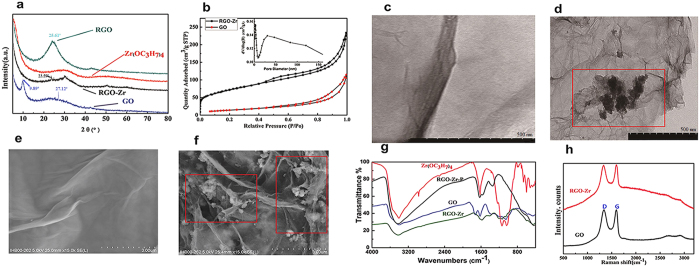
(**a**) XRD patterns of RGO-Zr, Zr(OC_3_H_7_)_4_, GO, and RGO. (**b**) Nitrogen adsorption–desorption isotherms of GO and RGO-Zr. (**c**) and (**d**) TEM images of GO and RGO-Zr. (**e**) and (**f**) SEM images of GO and RGO-Zr. (**g**) FTIR spectra of GO, Zr(OC_3_H_7_)_4_, RGO-Zr, and RGO-Zr after adsorption of phosphate. (**h**) Raman spectra of GO and RGO-Zr.

The XRD pattern of GO (Fig. 2a) showed a typical characteristic diffraction peak located at 2θ = 9.89° associated with the (001) inter-planar spacing of 0.89 nm, and another small peak at 2θ = 27.12° 27 induced by the graphite and assigned to d-spacing of 0.317 nm. In the pattern of RGO, the appearance of the (002) diffraction line at 2θ = 25.61° (d-spacing of 0.35 nm) and disappearance of the diffraction peak at 2θ = 9.89° demonstrated that GO was reduced to RGO^7^,^23^. Also, the small diffraction peaks of RGO-Zr (Fig. 2a) observed at 2θ = 23.59° (d-spacing of 0.38 nm) suggested the successful reduction of GO to RGO. The peak intensity of RGO-Zr decreased markedly compared to RGO and the inter-planar spacing rose from 0.35 (RGO) to 0.38 nm, suggesting that ZrO_2_ was mostly attached to the RGO surface layers leaving few intercalation between them. These data proposed that zirconium and GO were successfully combined to form a new composite called RGO-Zr through the hydrothermal method.

The nitrogen adsorption–desorption isotherms of GO and RGO-Zr (Fig. 2b) exhibited type IV isotherms, as defined by IUPAC classification^16^,^17^. A hysteresis loop of type H3 in RGO-Zr at the relative pressures [P/P_o_] ranging from 0.45 to 1.00 indicated the possible presence of mesoporous structure^17^,^18^. Figure 2b (inset) demonstrated that most pores of RGO-Zr were mesopores, corroborating the XRD results. The size distribution varied from 2 to 50 nm, where pores with smaller size attributed to the RGO-Zr particles while those with larger size represent the porosity in the aggregates. The BET surface areas, pore volumes, and average pore sizes of GO and RGO-Zr are presented in Table S1. It will be noted that RGO-Zr adsorbent had a relatively larger surface area, higher pore volume, and looser structure than that of GO. The increased porosity of RGO-Zr might be caused by exfoliation and rearrangement of the layers during the reduction process^17^. In other words, the use of RGO as a substrate is beneficial for dispersion of Zr(OC_3_H_7_)_4_ and enhancing the adsorption capability.

The TEM image of GO shown in Fig. 2c appeared transparent and folded over the edges. The SEM image of GO displayed typical crumplings and ripples on the surface (Fig. 2e), which could be induced by the multilayer scrolling of the GO sheets. These results are similar to those observed by other researchers^7^,^28^. The RGO-Zr (Fig. 2d) showed larger elastic corrugations and scrolled or folded edges than the GO (Fig. 2c), with ZrO_2_ particles attached to the surface of RGO. The changes in porosity of RGO-Zr were supported by the SEM images (Fig. 2f, Fig. S2), the porous and loose structure of RGO-Zr was detected. The ZrO_2_ particles were loaded on both the surface and edges of RGO, corresponding to changes in the inter-planar spacing of the XRD patterns. As a result, it was hard to distinguish the boundaries, which further demonstrates the combination of both materials.

Figure 2g displays the FTIR spectra of Zr(OC_3_H_7_)_4_, GO, RGO-Zr and RGO-Zr after adsorption of phosphate and the characteristic bands are listed in Table S2. The GO contains several functional groups C = O, C = C (C-C), O-C = O, O-H^17^,^29^, and Zr(OC_3_H_7_)_4_ have -C(CH3)_2_, -CH_3_, C = C, C-O-Zr, Zr-O groups^19^,^30^. For RGO-Zr, new peaks appeared at the bending vibrations of O-H groups mixed with Zr-OH bending^31^, the C = C skeletal vibration of the RGO sheets^32^, -CH_2_ symmetrical stretching vibrations, and -CH_2_ antisymmetric stretching vibration. Other peaks located at 1726 cm^−1^ (C = O) and 1380 cm^−1^ (O-C = O) were markedly diminished or vanished from the spectrum when compared with those of GO and Zr(OC_3_H_7_)_4_. After adsorption of phosphate, the O-H group (1055 cm^−1^) of RGO-Zr was significantly reduced in intensity or completely vanished from the spectrum, indicating that other groups interacted with the -OH groups^17^. Likewise, the characteristic band of C-O-Zr vibration was blue shifted and weakened and that of Zr-O became less pronounced, implying that Zr-OH groups served as the active sites for the phosphate adsorption^13^.

Raman spectroscopy is a powerful and nondestructive technique frequently used for characterization of the sp^2^ and sp^3^ hybridized carbon atoms present in carbonaceous materials^33^. It can be utilized to identify disorder and defect structures and distinguish mono-, bi-, and multi-layer characteristics of graphene materials^34^. The Raman spectrum of GO (Fig. 2h) displayed two bands at 1343 and 1600 cm^−1^, corresponding to the D and G modes, respectively^16^. The G-band was attributed to the in-plane vibration of sp^2^ bonded carbon atoms, while the D-band was associated with the presence of defects in the graphitic layers^16^,^35^. The spectrum of RGO-Zr (Fig. 2h) depicted a red-shift in the D-band (1340 cm^−1^) and G-band (1597 cm^−1^) when compared to GO. The ratio of D to G bands peak intensities (ID/IG) is a common index used to evaluate the extent of defects on GO and RGO^36^. The ID/IG ratio of RGO was estimated to 1.87 and thus higher than that of the pristine GO (1.33), suggesting the successful reduction of GO to RGO.

**Figure 3:**
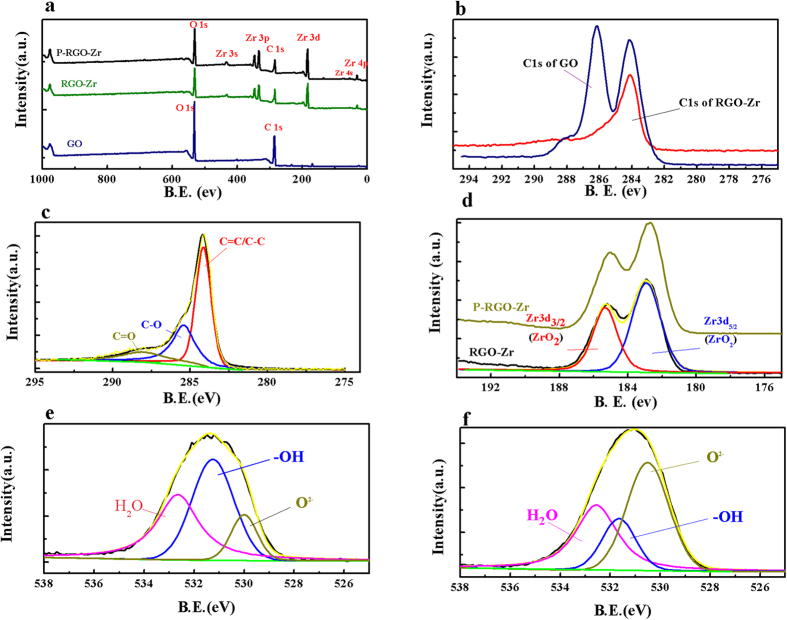
(**a**) XPS survey spectra of GO, RGO-Zr, and RGO-Zr after the phosphate adsorption. (**b**) The C1s binding energy of GO and RGO-Zr samples. (**c**) The C1s binding energy of RGO-Zr. (**d**) The Zr 3d binding energy of RGO-Zr and RGO-Zr after the phosphate adsorption. (**e**) The O 1 s binding energy of the RGO-Zr samples. (**f**) The O 1 s binding energy of RGO-Zr after adsorption of phosphate.

XPS allows the direct determination of the elemental composition of GO and RGO^27^. The full XPS scan (Fig. 3a) showed the elements present with their atomic percentages on GO, RGO-Zr, and RGO-Zr after the adsorption of phosphate (Table S3). The C/O ratio was increased from 1.33 for GO to 2.03 for RGO-Zr, suggesting a decrease in the oxygen groups during the reduction process. The percent of Zr atom at the surface of RGO-Zr was estimated to 8.45%. A computational multi-peak resolution method for XPS was applied to the C1s, Zr 3d, and an O 1 s band of RGO-Zr. The C1s band of RGO-Zr (Fig. 3c) can be deconvoluted into three peaks located at 284.14, 285.41 and 288.09 eV, corresponding respectively to the C = C/C-C, C-O and C = O groups^37^. These results were in accordance with the FTIR data that showed that C = O and O-C = O groups were markedly diminished or vanished from the materials. The spectrum of the core level of Zr 3d for RGO-Zr (Fig. 3d) revealed a strong spin–orbit doublet due to both the Zr 3d_5/2_ at 182.92 eV and Zr 3d_3/2_ at 185.32 eV. The XPS spectrum was constrained by the Zr 3d_5/2_–Zr3 d_3/2_ spin–orbit separation of 2.40 eV, and the ratio of the two peaks areas of each doublet was estimated to 3:2. These features are characteristic of Zr^4+^ ions in full oxidation states^38^,^39^. The Zr 3d binding energy increased after the adsorption of phosphate (Fig. 3d) due to the partial replacement of –OH groups by phosphate ion to form Zr-O-P linkage with lower negative charge density than Zr-O-H^7^. The O 1 s peak could be best fitted with three overlapped O 1 s peaks of oxide oxygen (O^2−^), a hydroxyl group (-OH), and adsorbed water (H_2_O). The fitting parameters could be found in Table S4. It is clear from Table S4, Fig. 3e and f that the –OH percentage in the total surface oxygen sharply dropped from 44.54% to 18.37% after the adsorption of phosphate. This demonstrated that hydroxyl groups were probably replaced during the adsorption of phosphate ion. Thus, the surface hydroxyl group played a key role in the adsorption of phosphate on the surface, which was in accordance with the FTIR findings.

**Figure 4:**
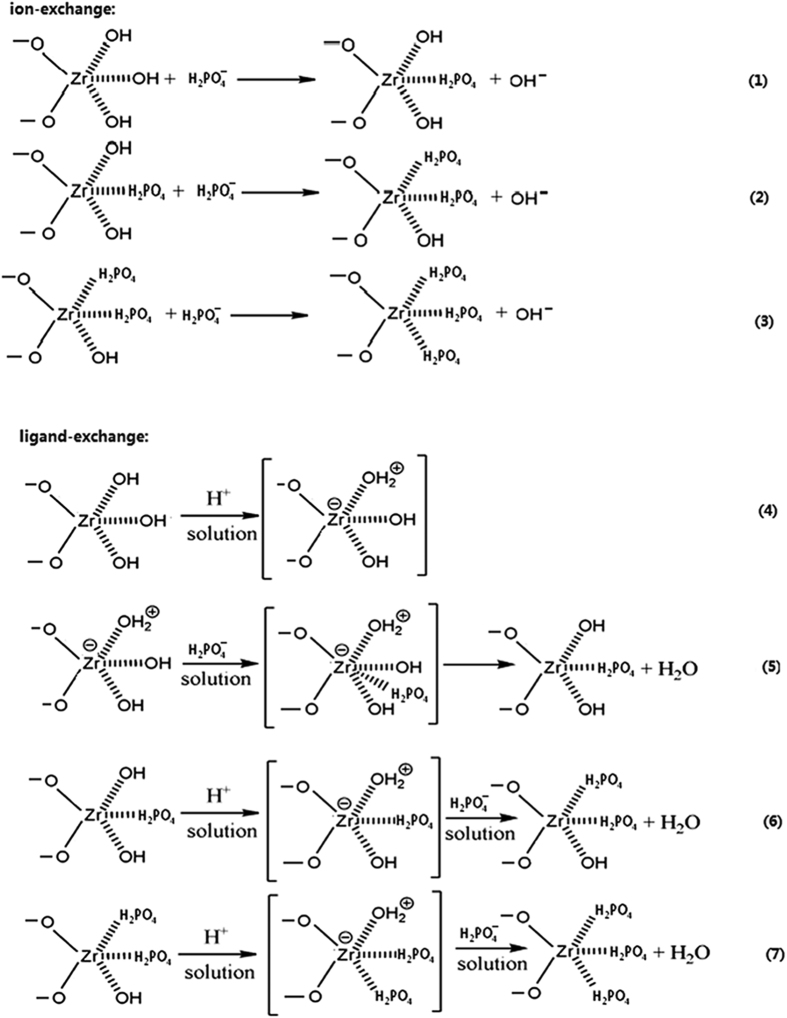
The main species of phosphate exits in weakly acidic solution and the possible reactions of phosphate adsorption on the surface of RGO-Zr.

Based on the above analyses and relevant literature^7^,^12^,^13^, the adsorption mechanisms of phosphate would contain ion exchange and ligand exchange (outer or inner sphere complexes). Given that phosphate exists in weakly acidic solution (pH = 5.00) as predominately HPO_4_^−^, thus the main reactions could be represented as in Fig. 4 7,12,13.

### Adsorption isotherms

The adsorption isotherms are useful to estimate the distribution of adsorbate molecules between the liquid and solid phases when the adsorption process reached an equilibrium state^40^. The adsorption capacity at various equilibrium concentrations of phosphate could also be revealed by the adsorption isotherms. Several mathematical models have been developed to describe the adsorption equilibria of phosphate on solid surfaces, such as the linear, Freundlich, and Langmuir models shown in equations (4) to (6). These are frequently utilized to fit the experimental data^1^.


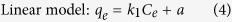



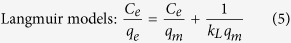



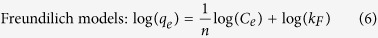


where *C*_*e*_ (mg/L) is the equilibrium concentration of phosphate in water, *q*_*e*_ (mg P/g) is the adsorption capacity of phosphate at equilibrium, *k*_*1*_ (L/g) is the slope of the linear model of phosphate adsorption efficiency, *a* (mg P/g) is the naturally adsorbed phosphate describing the adsorption of phosphate at low concentrations, *q*_*m*_ (mg P/g) is the maximum adsorption capacity of phosphate, *k*_*L*_ (L/mg) is the Langmuir adsorption isotherm constant related to the adsorption energy, *k*_*F*_ is the Freundlich adsorption constant related to the adsorption capacity, and *1/n* is an empirical parameter related to the intensity of adsorption.

**Figure 5:**
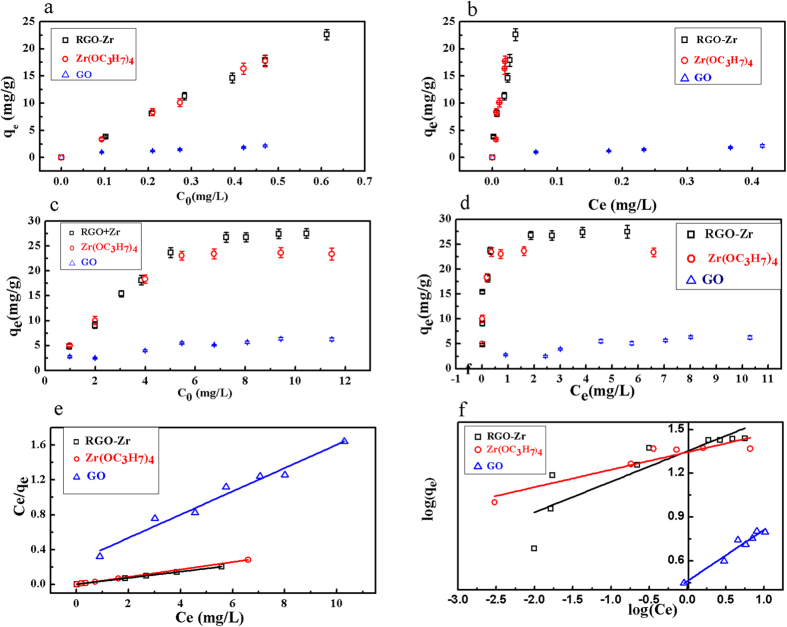
(**a**) and (**c**) Adsorption of phosphate on the RGO-Zr, Zr(OC_3_H_7_)_4_ and GO at different initial concentrations. (**b**) and (**d**) Adsorption isotherms of RGO-Zr, Zr(OC_3_H_7_)_4_, and GO. (**e**) Linearized Langmuir isotherm model. (**f**) Linearized Freundlich isotherm model for the adsorption of phosphate on the RGO-Zr, Zr(OC_3_H_7_)_4_, and GO.

Figure 5b demonstrates the adsorption isotherm of RGO-Zr, Zr(OC_3_H_7_)_4_ and GO at lower equilibrium concentrations of phosphate up to ~0.035 mg/L on RGO-Zr and Zr(OC_3_H_7_)_4_, and up to ~0.41 mg/L phosphate on GO. As shown in Table 1, the equilibrium data fit well with the Linear models. The three materials displayed different values of the parameter *a* (mg P/g) in the Linear models, and the highest value was recorded with RGO-Zr (*a* = 2.92). This means that compared to the others, RGO-Zr had an elevated adsorptivity for phosphate, which is naturally adsorbed at low concentrations of phosphate. Therefore, RGO-Zr composites can be used as a complementary technique to adsorb and treat the low concentrations of phosphate from the water after biological treatments.

The adsorption isotherms at elevated equilibrium concentrations of phosphate were compiled in Fig. 5c, and the parameters obtained by fitting the experimental data were summarized in Table 2. The results suggested that the equilibriums of the three materials could be better described by the Langmuir model (high values of R^2^) rather than by Freundilich model. Nevertheless, a value of 1/*n* < 0.5 in Freundilich model indicated a favorable adsorption and the strength of the adsorbent varied in the following order: Zr(OC_3_H_7_)_4_ > RGO-Zr > GO. The values of *q*_*m*_ estimated by the Langmuir models were 27.71 mg P/g, 23.42 mg P/g and 7.42 mg P/g for RGO-Zr, Zr(OC_3_H_7_)_4_ and GO, respectively. Although *q*_*m*_ might not an accurate estimation of a long-term adsorption capacity, it is still useful for comparing alternative materials towards adsorption of phosphate. The data revealed that adsorption of phosphate on the RGO-Zr was much higher than that of GO (Fig. 5a and c), suggesting an enhanced phosphate adsorption ability caused by the loaded ZrO_2_ on RGO-Zr.

Table 3 lists the various phosphate adsorbents and their adsorption capacities. Although the adsorption capacity of RGO-Zr was not the highest, it still advantageous because the synthetic method of RGO-Zr is green and does not add additional reagents or produce pollutants as the previously mentioned complicated and harmful methods. Beside, RGO was successfully obtained from GO in pure water without using any reductant or surfactant, and ZrO_2_ was tightly integrated on the RGO surface during the hydrothermal treatment. Furthermore, RGO-Zr could easily be applied for small-scale treatment facilities or wastewater with relatively low phosphate concentrations, while some reported phosphate adsorbents in the literatures^2^,^7^,^41^ have higher equilibrium concentrations of phosphate after treatment.

To evaluate the mechanisms involved in the adsorption process, the Gibbs free energy (ΔG) was calculated according to equations from the literature^17^ and the details are listed in Table S5. The obtained negative value of ΔG for RGO-Zr suggested that the adsorption was spontaneous and contained physisorption processes. Based on data, the RGO-Zr has a great potential for use as a phosphate adsorbent.

### Adsorption kinetics

**Figure 6:**
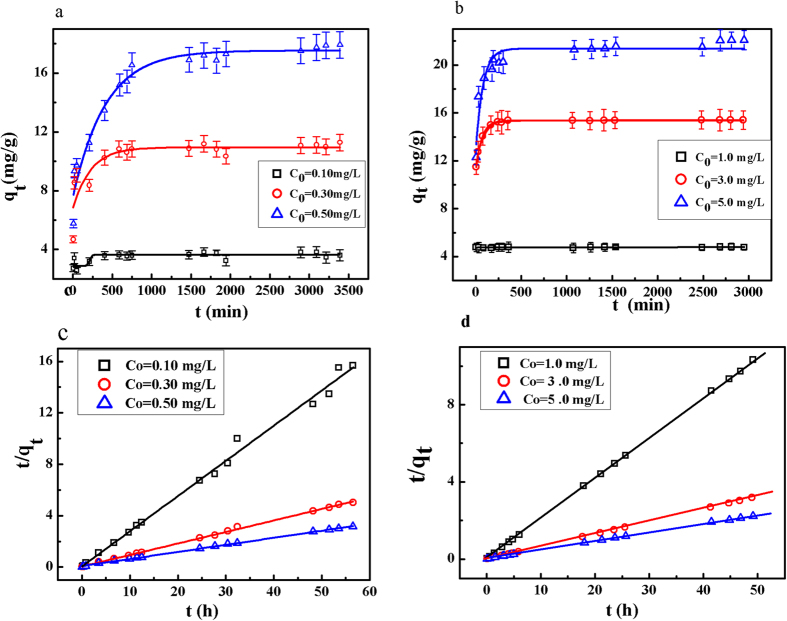
(**a**) Adsorption of phosphate on RGO-Zr at variable low initial phosphate concentrations (adsorbent dosage = 0.025 g/L, pH = 5.00 ± 0.20, temperature = 298 K). (**b**) Adsorption of phosphate on RGO-Zr at different high initial phosphate concentrations (adsorbent dosage = 0.20 g/L, pH = 5.00 ± 0.20, temperature = 298 K). The pseudo-second-order kinetic curve fitting for: (**c**) low initial phosphate concentrations and (**d**) high initial phosphate concentrations.

The kinetic curves of the adsorption of phosphate on RGO-Zr at variable initial phosphate concentrations are presented and compared in Fig. 6. As depicted in Fig. 6a and b, the adsorption process could be divided into two stages: a rapid adsorption at the early stage induced by a large number of the available adsorption sites for phosphate, then followed by a gradually slower stage over time until reaching the equilibrium. At the initial low phosphate concentrations of 0.10, 0.30 and 0.50 mg/L, the adsorption reached equilibrium within approximately 256, 438 and 650 min, respectively. However, at higher initial phosphate concentrations of 1.00, 3.00 and 5.00 mg/L, the adsorption reached equilibrium within 40, 250 and 375 min, respectively. The pseudo-first-order and pseudo-second-order equations were further used to model the kinetics data using a linear fitting, with the models expressed in equations (7) to (8) 2:


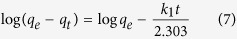



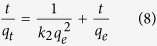


where *q*_*e*_ is the equilibrium adsorption amount (mg P/g), *q*_*t*_ is the adsorption amount (mg P/g) at any time *t* (h), and *k*_*1*_ (1/h) and *k*_*2*_ (g/(mg h)) are the pseudo-first-order and pseudo-second-order rate constants, respectively. The constant *k*_*2*_ is used to calculate the initial adsorption rate *h* (mg/(g h)) at t → 0 according to equation (9) 2:


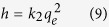


The so-called half-adsorption time *t*_*1/2*_ (h) represents the time required for the adsorption to take up half of phosphate as an equilibrium value, which is an indicator of the adsorption rate that could be calculated by equation (10) 2:


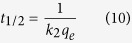


The kinetic model parameters obtained by fitting the experimental data are summarized in Table 4. The kinetic data could well be described by the pseudo-second-order kinetic model. The equilibrium adsorption amounts (*q*_*e,cal*_) calculated using the pseudo-second-order kinetic model were nearly identical to the experimental results (*q*_*e,exp*_), while the pseudo-first-order kinetic model did not fit well the data. This suggested that the adsorption process might be based on chemisorption, involving valence forces via shared or exchanged electrons between sorbent and sorbate (the replacement of -OH by phosphate)^6^,^31^. Similar kinetic results have been reported elsewhere for various phosphate adsorbents^1^,^2^,^6^. The overall rate of phosphate adsorption could be limited by the slower step, which would be either film diffusion or pore diffusion^8^. As the initial phosphate concentration increased and keeping other experimental conditions remained unchanged, the value of *k*_*2*_ decreased. This was induced by the numerous available adsorption sites at low initial phosphate concentrations if compared to high initial phosphate levels under the same experimental conditions.

At initial phosphate concentrations of 1.0, 3.0 and 5.0 mg L^−1^, the values of initial adsorption rate (*h*) were recorded as 135.14, 222.70 and 57.15 mg P/(g h) and the values of half-adsorption time (*t*_*1/2*_) were 0.04, 0.07 and 0.39 h, respectively. This suggested a declined adsorption rate and increased half-adsorption time as a function of the initial phosphate concentration at constant sorbent dose. The tendency was the same at the low initial phosphate concentrations but *h* and *t*_*1/2*_ were lower than those obtained with the initial high phosphate concentrations. Even so, the RGO-Zr could be considered as efficient for phosphate removal from aqueous solutions.

### Influence of pH, temperature and coexisting ions

**Figure 7:**
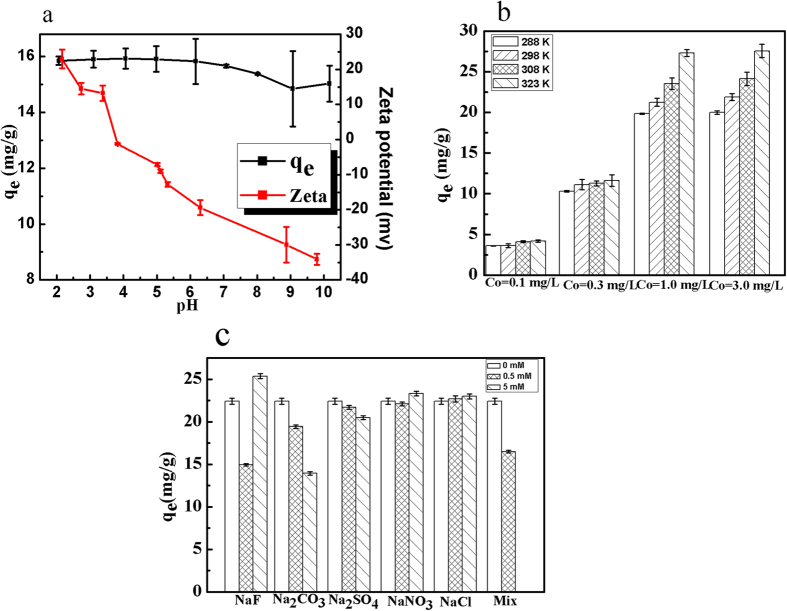
(**a**) Effect of pH on phosphate adsorption capacity of RGO-Zr and the Zeta potential of RGO-Zr (adsorption conditions: C_0_ = 5.00 mg/L, adsorbent dosage = 0.33 g/L, temperature = 298 K), (**b**) Effect of temperature on phosphate adsorption capacity of RGO-Zr (adsorption conditions: C_0_ = 0.10, 0.30, 1.00, 3.00 mg/L, adsorbent dosage = 0.025 g/L, pH = 5.00 ± 0.20, temperature = 288, 298, 308, 323 K), (**c**) Effect of coexisting anionic strengths on phosphate adsorption capacity of RGO-Zr (adsorption conditions: C_0_ = 5.00 mg/L, adsorbent dosage = 0.20 g/L, pH = 5.00 ± 0.20, temperature = 298 K).

The point of zero charge (pH_zpc_) of RGO-Zr estimated using zeta potential measurements was found to be around 3.62 (Fig. 7a). It is worth noting that pH = 5.00 is considered as optimum pH for its superior adsorption capacity compared to the others. Above pH_zpc_, the surface charge of RGO-Zr was negative. The surface charge of RGO-Zr could interfere with the adsorption of phosphate to some extent but never eliminates removal of phosphate in the entire pH range, where all removal efficiencies were found above 90%. The phosphate adsorption on RGO-Zr was found insensitive to the increase of pH, where electrostatic adsorption was probably not the main mechanism to explain the behavior. Thus, RGO-Zr can be applied for phosphate removal in natural waters with pH values between 6 and 9 with superior obtained results if compared to those requiring strict pH ranges^42^,^43^,^44^.

As shown in Fig. 7b, the adsorption capacity enhanced as the temperature increased, suggesting that phosphate adsorption on the RGO-Zr was endothermic. At the initial phosphate concentrations of 0.10 and 0.30 mg/L, the adsorption capacity enhanced slowly as the temperature rose. This phenomenon could be explained by the possibility that the adsorbent had absorbed nearly all the phosphate ions due to adequate adsorption sites of the adsorbent surface. Moreover, increased initial phosphate concentration resulted in enhanced adsorption capacity and adsorption efficiency, which could be attributed to the fact that higher temperatures can accelerate the diffusion of phosphate ions onto the adsorbent and enlarge the pore size of the adsorbent to some extent^45^.

Due to the complexity of substances present in natural waters, there might be competition in adsorption between the phosphate ions and other coexisting anions, such as SO_4_^2−^, F^−^, Cl^−^, NO_3_^−^ and CO_3_^2−^, which usually exist in natural waters and might affect the efficiency of phosphate adsorption. Figure 7c shows that SO_4_^2−^, F^−^, Cl^−^, NO_3_^−^, CO_3_^2−^ influence differently the phosphate adsorption capacities of RGO-Zr. The presence of Cl^−^, NO_3_^−^ in water showed slight or no effect on phosphate adsorption using RGO-Zr. However, the existence of SO_4_^2−^, CO_3_^2−^ lowered the phosphate adsorption on RGO-Zr. This could be related to the fact that Cl^−^ and NO_3_^−^ form only weaker bonds of outer sphere complexes, while SO_4_^2−^, CO_3_^2−^, HPO_4_^2−^ (H_2_PO_4_^−^, PO_4_^3−^) form both outer and inner sphere complexes with the active surface sites^8^. Thus, the different affinities of the coexisting anions to the adsorption sites could differently impact the phosphate adsorption. However, high concentrations of F^−^ could enhance the adsorption capacities, while low concentrations would not. This could be caused by the chemical reactions between ZrO_2_ and F^−^ 46. Considering the presence of much higher concentrations of the competing anions than the phosphate ions, these results clearly indicated that RGO-Zr still had good performance for removal of phosphate even at exceptionally elevated concentrations of competing ions. The latter was related to the relevant affinity of phosphate ions towards the adsorbent.

## Conclusions

RGO-Zr adsorbent was prepared by a simple and green hydrothermal method and demonstrated to be effective for phosphate adsorption in water in terms of adsorption capacity and speed. The characterization studies showed that GO was reduced to RGO, and the zirconium particles were successfully loaded on the RGO surface. Furthermore, the RGO-Zr displayed a porous structure and larger specific surface areas than GO. The equilibrium adsorption data of phosphate on RGO-Zr fitted well the Langmuir isotherm model with a maximum adsorption capacity recorded as 27.71 mg P/g at pH 5 and 298 K. The phosphate adsorption kinetics followed a pseudo-second-order kinetic model. Furthermore, the phosphate adsorption was found insensitive to the increase in pH, while the adsorption capacity enhanced as the temperature rose. The coexisting interfering anions SO_4_^2−^, F^−^, Cl^−^, NO_3_^−^ and CO_3_^2−^ exhibited different effects on the phosphate adsorption capacities of RGO-Zr. The mechanisms of the adsorption of phosphate on RGO-Zr probably involved ion exchange, ligand exchange and physical adsorption. With further optimization, the RGO-Zr sorbent has the potential to be used for controlling phosphorus pollution due to its superior properties.

## Additional Information

**How to cite this article**: Luo, X. *et al*. Adsorption of phosphate in water using one-step synthesized zirconium-loaded reduced graphene oxide. *Sci. Rep.* **6**, 39108; doi: 10.1038/srep39108 (2016).

**Publisher's note:** Springer Nature remains neutral with regard to jurisdictional claims in published maps and institutional affiliations.

## Supplementary Material

Supplementary Information

## Figures and Tables

**Table 1:** Linear models parameters for adsorption of phosphate on RGO-Zr, Zr(OC_3_H_7_)_4_ and GO at low initial phosphate concentrations.

Sample	*k*_*1*_ (L/g)	*a* (mg/g)	*R*^*2*^
RGO-Zr	533.64	2.93	0.9742**
Zr(OC3H7)4	837.12	1.06	0.9783**
GO	3.24	0.75	0.9752**

Note: ** means correlation analyses of the data by SPSS 19.0 resulted in equations with P < 0.01 (two sides), revealing the extremely significant correlation between *q*_*e*_ and *C*_*e*_.

**Table 2:** Langmuir and Freundlich isotherm models parameters for adsorption of phosphate on the RGO-Zr, Zr(OC3H7)4 and GO at high initial phosphate concentrations.

Sample	Langmuir model			Freundlich model		
	*q*_*m*_ (*mg*/*g*)	*k*_*L*_ (*L*/*mg*)	*R*^2^	*k*_*F*_	1/n	*R*^2^
RGO-Zr	27.71	17.19	0.9998**	22.56	0.21	0.8631**
Zr(OC_3_H_7_)_4_	23.42	85.40	0.9999**	24.02	0.15	0.9558**
GO	7.49	0.50	0.9734**	2.73	0.35	0.9511**

Note: ** means correlation analyses of the data by SPSS 19.0 resulted in equations with P < 0.01 (two sides), and revealing the extremely significant correlation between *q*_*e*_ and C_e_.

**Table 3:** Phosphate adsorption capacities of various adsorbents.

Adsorbent	pH	Adsorption capacity (mg/g)
Fe-Mn binary oxide^41^	5.60	33.20
Fe-Zr binary oxide^14^	5.50	102.30
ZrMZ^2^	7.00	10.20
Mesoporous ZrO_2_^13^	6.80	29.71
Zr-SBA-15^15^	6.00	13.77
Innovative modified bentonites^9^	7.00	11.15
am-ZrO_2_ nanoparticles^6^	6.20	99.01
Amorphous Zr(OH)_2_^12^	no data	17.00
Zr-Mg-Al^42^	8.70	30.00
Ferric sludge^47^	5.50	25.50
GO-Zr^7^	6.00	16.45
Lanthanum-doped activated carbon fiber^48^	no data	22.86
Iron-doped activated carbon^43^	3.78	14.12
RGO-Zr (Present study)	5.00	27.71

**Table 4:** The kinetic models parameters for phosphate adsorption on the RGO-Zr at pH 5.00 ± 0.20 and 298 K.

*C*_0_ (*mg*/*L*)	*q*_*e,*exp_ (*mg*/*g*)	Pseudo-first-order kinetic model			Pseudo-second-order kinetic model		
		*q*_*e,cal*_ (*mg*/*g*)	*k*_1_ (1/*h*)	*R*^2^	*q*_*e,cal*_ (*mg*/*g*)	*k*_2_ (*g*/mg*h*)	*R*^2^
0.10	3.60	1.64	0.25	0.8123	3.65	1.40	0.9939
0.30	11.11	4.81	0.31	0.6417	11.15	0.19	0.9989
0.50	17.89	11.52	0.14	0.9044	18.08	0.05	0.9987
1.00	4.78	1.34	0.45	0.6670	4.79	5.89	0.9999
3.00	15.36	4.22	0.93	0.9963	15.43	0.95	1.0000
5.00	22.08	6.94	1.94	0.9318	22.07	0.11	0.9997
