# Supplementary material for: Adsorption of phosphate in water using one-step synthesized zirconium-loaded reduced graphene oxide
Source: Sci Rep. 2016 Dec 15;6:39108. doi: 10.1038/srep39108 (PMC5156910; doi:10.1038/srep39108)
Supplement: Supplementary Information [file srep39108-s1.pdf]

Supplementary Informations

Adsorption of phosphate in water using one-step synthesized zirconium-loaded reduced graphene oxide

Xin Luo[1, 2], Xiurong Wang[1], Shaopan Bao[1, 2], Xiawei Liu[1, 2], Weicheng Zhang[1], Tao Fang[*1]

[1]Institute of Hydrobiology, Chinese Academy of Sciences, Wuhan 430072, China

[2]Graduate University of Chinese Academy of Sciences, Beijing 100049, China

*Corresponding author Emails: fangt@ihb.ac.cn

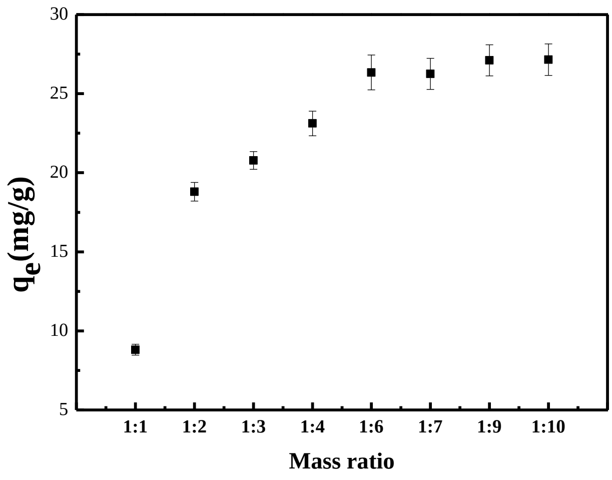

Fig. S1 The phosphate adsorption capacities of the composites with different mass ratios of GO : $Zr(OC_3H_7)_4$ (C_0 = 4.70 mg/L, T = 298 K, pH = 5.00 ± 0.20, adsorbent dosage = 0.10 g/L).

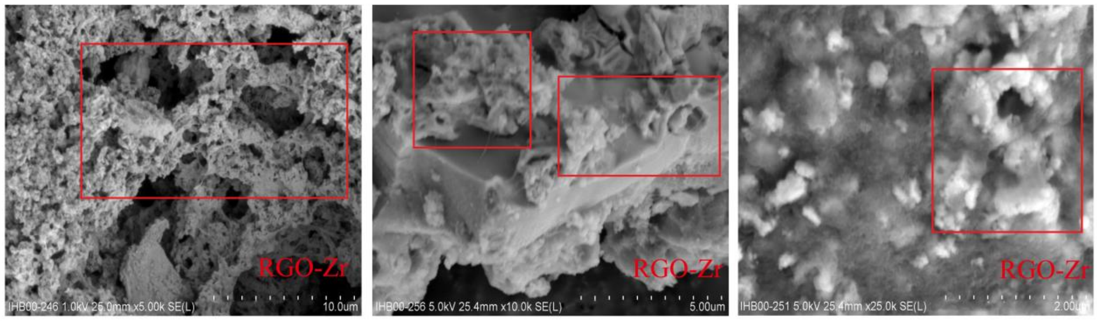

Fig. S2 TEM images of RGO-Zr.

Sample	GO	RGO-Zr
S_{BET} (m^2/g)	46.00	251.10
Pore volume (cm^3/g)	0.16	0.28
Average pore size (nm)	1.41	5.55

Table S1 BET data

Sample	Peak (cm^{-1})	group
GO	1726	C=O stretching of carboxylic groups
	1625	C=C (C-C) skeletal vibration
	1376	O-C=O groups vibrations
	1210	epoxy symmetrical ring deformation vibrations
	1160	O-H bend/stretch
	1057	C–O stretching vibrations mixed with C–OH bending
	3404	O-H stretching vibrations came from adsorbed and inhibited water
$Zr(OC_3H_7)_4$	2973	$-CH_3$ stretching vibration
	1621	C=C aromatic vibrations
	1365,1380	$-C(CH3)_2$
	1039	C-O-Zr vibrations
	952	
	400- 600	bridging-hydroxy perpendicular-to-plane Zr-O stretch
RGO-Zr	1055	bending vibrations of O-H mixed with Zr-OH bending
	1580	C=C skeletal vibration of the RGO sheets
	2921	$-CH_2$ symmetrical stretching vibrations
	2848	$-CH_2$ antisymemetric stretching vibration

Table S2 FTIR spectrum of GO, $Zr(OC_3H_7)_4$, RGO-Zr

Sample	Peak	Position (ev)	FWHM	Percent (%)
GO	C 1S	286.14	1.30	55.06
	O 1S	531.83	1.91	41.54
	S 2P	169.40	2.50	3.40
RGO-Zr	C 1S	284.12	1.36	59.02
	O 1S	531.30	3.32	29.03
	Zr 3d	183.05	2.13	8.45
	S 2P	169.47	2.51	3.50
phosphate/RGO-Zr	C 1S	284.08	2.13	50.96
	O 1S	531.13	3.22	38.07
	Zr 1S	182.90	2.23	8.55
	S 2P	170.00	2.45	2.42

Table S3 The XPS parameters for GO, RGO-Zr before and after phosphate adsorption.

Sample	Peak	Position	Area	FWHM	Percent (%)
RGO-Zr	H_2O	532.65	44451.75	2.00	42.21
	-OH	531.25	46897.31	1.98	44.54
	O^{2-}	530.00	13959.84	1.22	13.25
phosphate/RGO-Zr	H_2O	532.55	38795.62	2.00	37.13
	-OH	531.65	19195.85	1.59	18.37
	O^{2-}	530.49	46507.64	1.99	44.50

Table S4 The Lorentzian peak shape fitting parameters for O 1s XPS peak of RGO-Zr before and after phosphate adsorption.

Samples	T (K)	ΔG^0 (kJ/mol)
RGO-Zr	298	-7.05
$Zr(OC_3H_7)_4$	298	-11.02
GO	298	1.72

Table S5 Thermodynamic parameters for the phosphate adsorption on the RGO-Zr, $C_{12}H_{28}O_4Zr$ and GO
